# An Extensive Analysis of *Artemisia integrifolia* Linn. on T2DM: Investigating Glycolipid Metabolism, Metabolic Profiling, and Molecular Docking for Potential Functional Food Applications

**DOI:** 10.3390/foods14172945

**Published:** 2025-08-24

**Authors:** Meng Liu, Fazhi Su, Yujia He, Minghao Sun, Chenxi Bai, Wensen Zhang, Biao Li, Yanping Sun, Qiuhong Wang, Haixue Kuang

**Affiliations:** 1 Key Laboratory of Basic and Application Research of Beiyao, Heilongjiang University of Chinese Medicine, Ministry of Education, Harbin 150040, China; liumeng1710@163.com (M.L.); sfz18406564303@163.com (F.S.); heyujia125@163.com (Y.H.); m13125531353@163.com (M.S.); bcx19990709@163.com (C.B.); zhang1033362077@163.com (W.Z.); 18826108664@163.com (B.L.); sunyanping@hljucm.edu.cn (Y.S.); 2 School of Traditional Chinese Medicine, Guangdong Pharmaceutical University, Guangzhou 510006, China

**Keywords:** *Artemisia integrifolia* Linn., glycolipid metabolism, metabolic profiling, S1P, PI3K/AKT signaling pathway, T2DM

## Abstract

Type II diabetes mellitus (T2DM) is characterized by chronic glycolipid metabolic dysregulation. This study aimed to investigate the effects and mechanisms of *Artemisia integrifolia* Linn. (LH) as a functional food in a T2DM rat model. The UPLC-Q-TOF-MS/MS technique was used to identify the components of LH. T2DM was induced in rats via a high-fat/high-sugar diet combined with streptozotocin (STZ, 35 mg/kg, i.p.). The rats were subsequently treated with LH (90 mg/kg, 180 mg/kg) for 15 days. A total of 66 compounds were identified in both positive and negative ions. LH treatment resulted in an increase in body weight while reducing FBG levels. It also improved insulin resistance, blood lipid levels, liver pathology, function, and lipid accumulation. Furthermore, 18 metabolites and 5 metabolic pathways were identified in the liver. Mechanistically, LH may improve T2DM through modulation of the S1P and PI3K/AKT signaling pathway. Caffeic acid, coumarin, trifolin, and apigetrin were identified as the likely active components. In conclusion, LH may mitigate glycolipid metabolism disorders in T2DM rats by modulating metabolic profiling, S1P, and the PI3K/AKT signaling pathway, supporting its potential as a functional food.

## 1. Introduction

Diabetes mellitus (DM) is a chronic metabolic dysfunction characterized by dysregulation of glucose and lipid homeostasis. In 2021, the prevalence of DM has reached 10.5% [1]. It has emerged as the third most prevalent non-communicable chronic disease and can pose a significant threat to public health [2]. DM is conventionally categorized into type I diabetes mellitus (T1DM) and type II diabetes mellitus (T2DM) [3]. T2DM, representing 90–95% of diabetes cases, arises from a combination of genetic predisposition and environmental influences and is characterized by insulin resistance [4]. The liver is essential in maintaining glucose homeostasis by storing and releasing glucose [3]. Additionally, impaired hepatic lipid metabolism can aggravate diabetic liver damage, underscoring the liver’s pivotal role in glycolipid metabolism [5]. Excessive energy intake can lead to abnormal lipid accumulation in hepatic cells when the liver’s metabolic capacity is exceeded [6]. Hence, it is critical to identify therapeutic agents that can improve glycolipid metabolism in the treatment of T2DM.

Metabolomics involves the comprehensive identification and quantification of low-molecular-weight compounds within biological matrices to characterize metabolic phenotypes [7]. This methodology enables absolute quantification of organism-wide metabolite profiles while establishing correlations between metabolic dynamics and phenotypic variations under both normal and disease conditions. It aids in elucidating the organism’s pathophysiological condition and can reveal biomarkers of disease. In recent years, metabolomics has become increasingly important in studying metabolic diseases, providing a novel framework for assessing the overall efficacy of therapeutic interventions.

Sphingosine-1-phosphate (S1P), an important bioactive lipid, plays multiple roles by influencing the PI3K/AKT signaling pathway [8], which is crucial for glucose uptake and lipid metabolism during the development of T2DM [9]. This pathway plays a pivotal role in managing hyperglycemia in T2DM by regulating glucose uptake and glycogen synthesis. In the context of T2DM, the activation of PI3K promotes the translocation of AKT, which subsequently activates downstream receptors, leading to various physiological effects [9]. As a central metabolic regulator, AKT promotes cell surface glucose transporter protein 4 (GLUT4) translocation, thereby improving hepatocellular glucose uptake capacity and mitigating diabetic pathophysiology [10]. Moreover, GLUT2 is the primary glucose transporter isoform in hepatic tissues, playing a crucial role in mediating intracellular glucose uptake and facilitating hepatocellular glucose clearance [11]. Therefore, therapeutic targeting of the S1P and PI3K/AKT pathway may offer a promising approach for T2DM intervention, encompassing both disease prevention and clinical management.

Currently, commonly prescribed hypoglycemic medications for the clinical management of T2DM include incretin analogues, sodium-glucose cotransporter inhibitors, and insulin injections [12]. However, these treatments cannot fully satisfy the needs of T2DM patients, and prolonged use may result in various side effects. Therefore, it remains crucial to identify new effective drugs for the treatment of T2DM. Functional foods, known for their low toxicity and high efficiency, are increasingly being explored as treatment options for T2DM. *Artemisia integrifolia* Linn. (Chinese name: Liuhao, LH), a functional food with multiple medicinal properties and biological activities, is widely distributed in Northeast China. According to the Compendium of Materia Medica, LH is noted for its anti-inflammatory, antipyretic, liver-nourishing, and antihypertensive effects. Modern pharmacological investigations have additionally confirmed its multi-target bioactivities, including hypolipidemic effects, hepatic protection, and oxidative stress mitigation [13]. For example, studies have confirmed that LH has a good hypolipidemic effect [14]. Moreover, LH contains a variety of bioactive ingredients, including flavonoids, phenolic acids, and volatile oils [13,15]. Research suggested that Artemisia species may hold potential as candidates for managing diabetes [16]. In fact, in northeastern China, there is a folk custom of consuming the twigs of LH to treat diabetes. However, few studies have deeply reported the therapeutic effects of LH on T2DM. Therefore, this study elucidated the therapeutic prospects of functional foods for T2DM management while establishing a foundation for subsequent development of nutraceutical interventions.

## 2. Materials and Methods

### 2.1. Materials

Metformin (Met, 0.85 g/piece, 30 pcs/box, Y13618) was obtained from Merck & Co., Inc. (Rahway, NJ, USA). High-fat and high-sugar (HFHS) feed (SCXK (Beijing) 2023-0010) was sourced from Xiaoshu Youtai Technology Co., Ltd. (Beijing, China). STZ (2231030006) was purchased from Solarbio Technology Co., Ltd. (Beijing, China). The assay kits used for the measurement of glucose (GLU, 20231107), alanine aminotransferase (ALT, 20230420), aspartate aminotransferase (AST, 20230625), alkaline phosphatase (ALP, 20230214), triglycerides (TG, 20231220), cholesterol (CHO, 20220928), low density lipoprotein cholesterol (LDL-C, 20230720), and high density lipoprotein cholesterol (HDL-C, 20230718) were obtained from Biosino Bio-Technology and Science Inc. (Beijing, China). Primary antibodies for PI3K (Cat# 60225-1-Ig), GLUT2 (Cat#66889-1-Ig), and GLUT4 (Cat# 66846-1-Ig) were purchased from Proteintech Group, Inc. (Wuhan, China). Antibodies for AKT (Cat# A18675) and p-AKT (Cat# AP1453) were acquired from ABclonal Technology Co., Ltd. (Wuhan, China). The p-PI3K (bs-5570R) antibody was provided by Bioss Co., Ltd. (Beijing, China).

### 2.2. Preparation and Characterization of LH

The aerial parts of *Artemisia integrifolia* Linn. (LH) were harvested from Qiqihar City, Heilongjiang Province, China, in May 2023. The plant material was authenticated by Professor Lianjie Su of Heilongjiang University of Traditional Chinese Medicine and stored at the university’s Department of Traditional Chinese Medicine Chemistry. The aerial parts of freshly collected *Artemisia integrifolia* Linn. were air-dried naturally, cut into 3–5 cm segments, and uniformly mixed with leaves before weighing. Approximately 2 kg of LH powder was extracted three times with 75% ethanol at a 1:10 (g:mL) ratio for 3 h each. The extract was concentrated under reduced pressure and dried, yielding an extraction rate of 40.25%.

For component analysis, 5 mg of LH extract was accurately weighed and mixed with methanol:water (2:1, *v*/*v*). Then it was homogenized, vortexed, and centrifuged. The chromatographic analysis was conducted on a Thermo Scientific™ (Thermo Scientific, Waltham, MA, USA) Vanquish UHPLC platform equipped with a Hypersil Gold Vanquish column (2.1 × 100 mm, 1.9 μm). Separation conditions included a column temperature of 30 °C and a mobile phase flow rate of 0.3 mL/min. The gradient program utilized mobile phase A (0.1% aqueous formic acid) and phase B (0.1% formate in acetonitrile) with the following profile: 0–2 min, 90% A; 2–10 min, 90–50% A; 10–10.1 min, 50–20% A; 13–14 min, 20–5% A; 14–14.1 min, 5–90% B; 14.1–18 min, 90% A. Mass spectrometer detection was performed on a Thermo Scientific^TM^ Q ExtractiveTM Plus (spray voltage: 3.5 kV/+ and 3.2 kV/−; capillary temp: 320 °C; auxiliary gas heater temp: 350 °C).

### 2.3. Animal Experiment

Eight-week-old male SD rats (180–220 g) were procured from Liaoning Changsheng Biotechnology Co., Ltd. (Shenyang, China, SYXK (Liao) 2020-0001). The experimental protocol was approved by the Laboratory Animal Ethics Committee of Heilongjiang University of Chinese Medicine (Approval NO. 2024062001, approve date: 20 June 2024). The animals were maintained in SPF facilities at 25 ± 2 °C with 50–60% relative humidity under 12 h light/dark cycles, with free access to feed and water. After 1-week acclimation, 8 rats were randomly assigned to the normal control (CON) group and fed a standard diet. The remaining rats were given a HFHS feed (66.5% rat maintenance feed, 20% sucrose, 2.5% cholesterol, 10% lard, and 1% sodium cholate). After 5 weeks, they were injected intraperitoneally with STZ (35 mg/kg in 0.1 mol/L citrate buffer, pH 4.5) after 12 h of fasting and water deprivation. Control rats were administered citrate buffer in equivalent volumes. Rats displaying fasting blood glucose (FBG) <11.1 mmol/L received a subsequent STZ dose. Diabetes induction was verified 72 h after the final injection by confirmed hyperglycemia (FBG >8 mmol/L), along with typical diabetic symptoms (polydipsia, polyphagia, polyuria, and weight loss). A total of 32 rats met these criteria.

### 2.4. Intervention of Drugs, Collection of Blood and Liver Tissues

T2DM model rats were divided into four groups based on FBG levels randomly: model (M), metformin (Met, 200 mg/kg), low-dose LH (LH-L, 90 mg/kg), and high-dose LH (LH-H, 180 mg/kg). The dosage of LH relies on previously published studies [14]. The CON group continued on a standard diet, while all other groups remained on the HFHS diet. After 15 days of drugs and once-daily intervention, rats were fasted and water-deprived. Blood was obtained through abdominal aortic puncture followed by centrifugation (3000 rpm, 10 min). Hepatic tissues were excised and preserved at −80 °C along with serum samples.

### 2.5. Body Weight and FBG

Body weights were recorded weekly. FBG was measured at baseline (day 0) and on days 4, 7, 9, 12, and 15 post-treatment, after 6 h of fasting and water deprivation.

### 2.6. Histopathological Analysis

Following 4% paraformaldehyde fixation, liver samples were embedded in paraffin and cut into 5-µm sections for microscopic evaluation. Histological evaluation included H&E staining for morphological assessment and steatosis detection, along with Oil red O (ORO) staining of cryopreserved specimens for lipid visualization. Images were captured using a Nikon Eclipse Ci-L upright microscope (Nikon, Tokyo, Japan).

### 2.7. Biochemical Assays

Serum levels of GLU, ALT, AST, ALP, TG, CHO, HDL-C, and LDL-C were determined using assay kits. Liver tissues (100 mg) were homogenized in 900 µL of saline at 4 °C, followed by centrifugation at 5000× *g* for 10 min. The supernatant was used to measure S1P levels according to the kit instructions.

### 2.8. Insulin and Insulin Resistance Index

The levels of fasting insulin (FINS) in rat serum were determined using an ELISA kit (Lot: 202505) from Jiangsu Enzyme Free Industrial Co., Ltd. (Nanjing, China), and homeostatic model assessment for insulin resistance (HOMA-IR) was calculated as FINS (mU/L) × FBG (mmol/L)/22.5.

### 2.9. Untargeted Metabolomics Analysis

For metabolomic profiling, 100 mg hepatic tissue aliquots were equilibrated at 4 °C and extracted with 600 μL solvent mixture (methanol: acetonitrile: water, 2:2:1 *v*/*v*/*v*). Following homogenization and vortexing, samples were centrifuged (14,000 rpm, 10 min, 4 °C). A pooled QC sample was generated by mixing equal 20 μL volumes of supernatant from all individual specimens. System conditioning involved six replicate QC injections prior to analytical runs, with intermittent QC analysis (every sixth sample) for continuous performance monitoring during UPLC-Q-TOF-MS/MS operation.

The analytical platform comprised a Waters SYNAPT G2-Si Q-TOF mass spectrometer interfaced with an ACQUITY UPLC system. An HSS T3 column (2.1 × 100 mm, 1.8 μm particles) maintained at 35 °C with 0.3 mL/min mobile phase flow was used for the separation. The gradient elution program was as follows: phase A (0.1% aqueous formic acid) and phase B (acetonitrile containing 0.1% formic acid). The gradient was set as 0–9 min, 95–40% A; 9–11 min, 40–5% A; 11–12 min, 95% A; 12–14 min, 95% A. The autosampler temperature was maintained at 10 °C, and each sample was injected at a volume of 2 μL. Mass spectrometry detection was conducted in both negative and positive electrospray ionization source (ESI) modes, with the following mass conditions: cone voltage of 40 V, collision energy of 20–35 V, and a scanning range of *m*/*z*, 100-1, 200.

The raw mass spectrometry data were imported into Progenesis QI software. PCA and OPLS-DA were performed using the SIMCA-P platform. The model fitness was evaluated using the R2Y and Q2Y values. Metabolites of interest were identified based on VIP scores from the OPLS-DA model and the *p*-value derived from Student’s *t*-test. Candidate biomarkers were identified through dual criteria (VIP > 1.0, *p* < 0.05). Metabolic pathway analysis was conducted utilizing HMDB, MetaboAnalyst 6.0, and KEGG platforms [17]. Additionally, the Metware Metabolic Cloud Platform (https://cloud.metware.cn/#/home, accessed on 1 February 2024) was used for Spearman correlation analysis between biochemical indexes and liver metabolites.

### 2.10. Quantitative Real-Time PCR (RT-qPCR)

Total RNA was extracted from liver tissues using Trizol reagent (DP424, Tiangen, Beijing, China). RNA was reverse transcribed into cDNA using the RevertAid First Strand cDNA Synthesis Kit (Thermo, Waltham, MA, USA). Primers for target rat genes were synthesized by Jinweizhi (Beijing, China), as listed in Table 1. RT-qPCR was performed on a Veriti real-time PCR system (ABI, Los Angeles, CA, USA). Relative fold change was calculated using the 2^−∆∆Ct^ method and normalized to GAPDH.

### 2.11. Immunofluorescence Staining

Liver tissue sections were prepared as described in the histopathological analysis section. The slices were deparaffinized, rehydrated, blocked, and then incubated overnight at 4 °C with PI3K, p-PI3K, AKT, p-AKT, GLUT4, and GLUT2 (1:200, 1:200, 1:500, 1:200, 1:200, and 1:500) primary antibodies. The slices were incubated with secondary antibodies and DAPI the next day. The slices were then mounted and scanned using a digital slide scanner.

### 2.12. Molecular Docking

Molecular docking was performed as previously described [18]. The small molecules were caffeic acid, coumarin, trifolin, and apigetrin, with PI3Ka (8V8V) serving as the protein receptor.

### 2.13. Statistical Analysis

Statistical evaluations were performed in SPSS 26.0 (IBM, Los Angeles, CA, USA) with results expressed as mean ± SD. Student’s *t*-test analyzed pairwise differences, whereas multi-group comparisons utilized one-way ANOVA, considering *p* < 0.05 statistically significant.

## 3. Results

### 3.1. Determination of the Chemical Components of LH

UPLC-Q-TOF-MS/MS analysis enabled comprehensive characterization of LH constituents (Figure S1, Tables S1 and S2). The profile comprised 40 and 26 compounds detected in positive and negative ionization modes, respectively, predominantly flavonoids, organic acids, and coumarin derivatives. The major components of LH included flavonoids, acids, and coumarins. To identify the potential active components of LH, we focused on compounds with relatively high peak intensity. These included caffeic acid, coumarin, trifolin, and apigetrin, which were selected for further investigation.

### 3.2. Effects of LH on FBG, Body Weight, Insulin, and HOMA-IR in T2DM Rats

**Figure 1:**
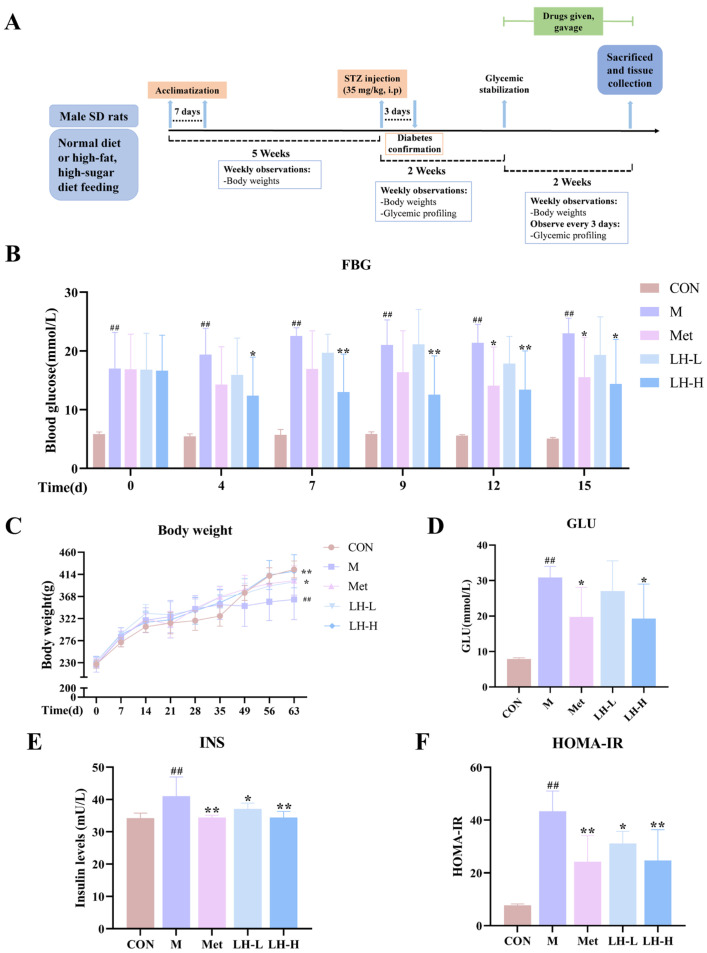
Effects of LH on FBG and body weight in T2DM rats. (**A**) Experiment design and protocol. (**B**) FBG levels during drug intervention (n = 8). (**C**) Changes in body weight (n = 8). (**D**) Serum glucose levels (n = 6). (**E**) Serum insulin levels (n = 6). (**D**) HOMA-IR levels (n = 6). CON, control group; M, T2DM model group; Met, metformin group; LH-L, 90 mg/kg LH group; LH-H, 180 mg/kg LH group. ^##^ *p* < 0.01 vs. CON group; * *p* < 0.05, ** *p* < 0.01 vs. M group.

Significant body weight reduction and elevated FBG levels were exhibited in the model group (Figure 1, *p* < 0.01). Following drug intervention, metformin and high-dose LH (LH-H) treatment significantly increased body weight (*p* < 0.05 or *p* < 0.01) and lowered FBG and serum glucose levels (*p* < 0.05 or *p* < 0.01) in T2DM rats. Moreover, metformin and LH decreased the levels of insulin and HOMA-IR (*p* < 0.05 or *p* < 0.01) in T2DM rats.

### 3.3. Effects of LH on Hepatic Morphology, Steatosis, and Function in T2DM Rats

**Figure 2:**
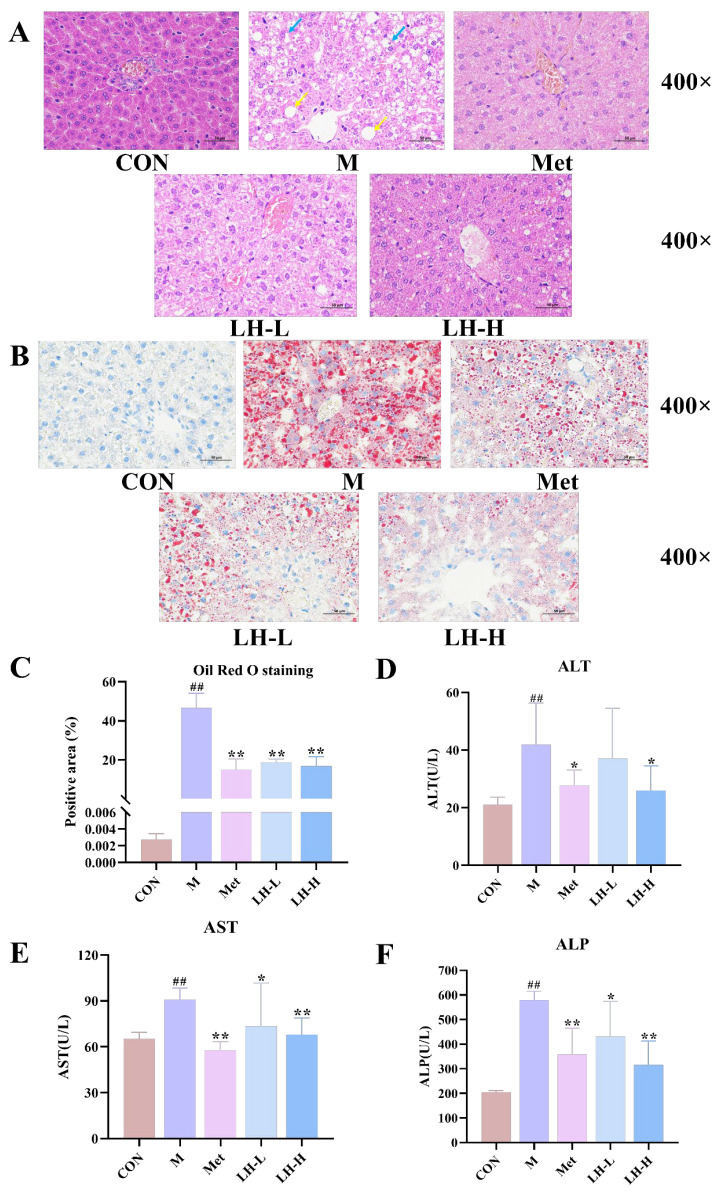
Effects of LH on hepatic morphology, steatosis, and function in T2DM rats. (**A**–**C**) H&E and ORO staining as well as quantitative analysis of liver (×400; n = 3). (**D**–**F**) ALT, AST, and ALP levels in rat serum (n = 6). Scale bar = 50 µm. Blue arrows indicate hepatocellular watery degeneration; yellow arrows denote hepatic steatosis. ^##^ *p* < 0.01 vs. CON group; * *p* < 0.05, ** *p* < 0.01 vs. M group.

As depicted in Figure 2, H&E-stained liver sections from the control group exhibited characteristic hepatocyte architecture with well-defined cellular margins and no lipid accumulation, indicating preserved hepatic histology. In contrast, the model group exhibited extensive hepatocellular watery degeneration, prominent central venous congestion, and mild hepatic steatosis. Both metformin and LH treatment (LH-L and LH-H) ameliorated liver pathological damage. ORO staining revealed a significant reduction in liver lipid droplet accumulation following drug treatments (*p* < 0.01). Additionally, liver function markers, including ALT, AST, and ALP, were significantly elevated in the T2DM group (*p* < 0.01). After drug intervention, the serum activities of these enzymes were significantly reduced (*p* < 0.05 or *p* < 0.01). These findings confirmed that LH had hepatoprotective effects and ameliorated liver function impairment and lipid accumulation in T2DM rats.

### 3.4. Effects of LH on Dyslipidemia in T2DM Rats

**Figure 3:**
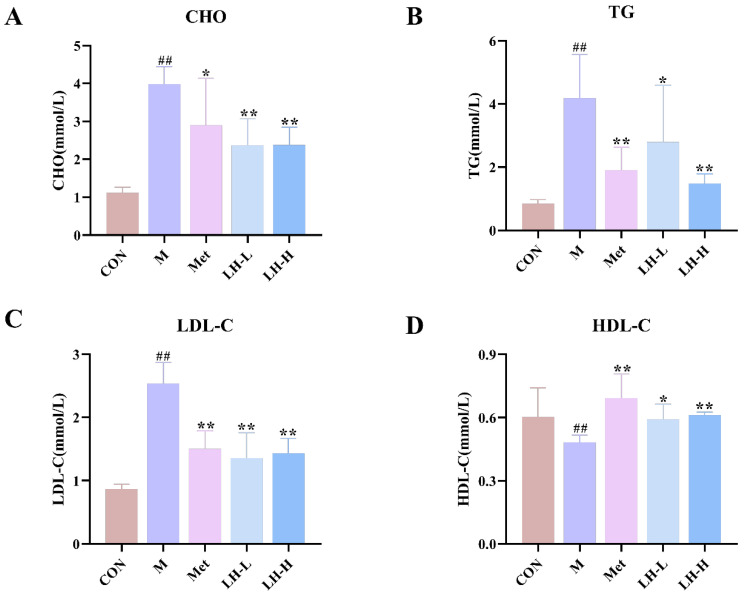
Effects of LH on dyslipidemia in T2DM rats. (**A**–**D**) Serum levels of CHO, TG, LDL-C, and HDL-C (n = 6). ^##^ *p* < 0.01 vs. CON group; * *p* < 0.05, ** *p* < 0.01 vs. M group.

The T2DM group exhibited markedly elevated serum levels of CHO, TG, and LDL-C (*p* < 0.01), along with significantly reduced HDL-C levels (*p* < 0.01) compared with the control group. After drug intervention, CHO, TG, and LDL-C levels were reduced to varying extents, while HDL-C levels increased (*p* < 0.05 or *p* < 0.01). These findings demonstrated that LH may effectively improve lipid metabolism disorders in T2DM rats (Figure 3).

### 3.5. Effects of LH on Liver Metabolism in T2DM Rats

An untargeted metabolomics analysis of liver tissues was employed by UPLC-Q-TOF-MS/MS technology. Compounds detected in ESI+ and ESI- ionization modes were subjected to multivariate statistical analysis. The QC samples clustered together, demonstrating good reproducibility and system stability.

**Figure 4:**
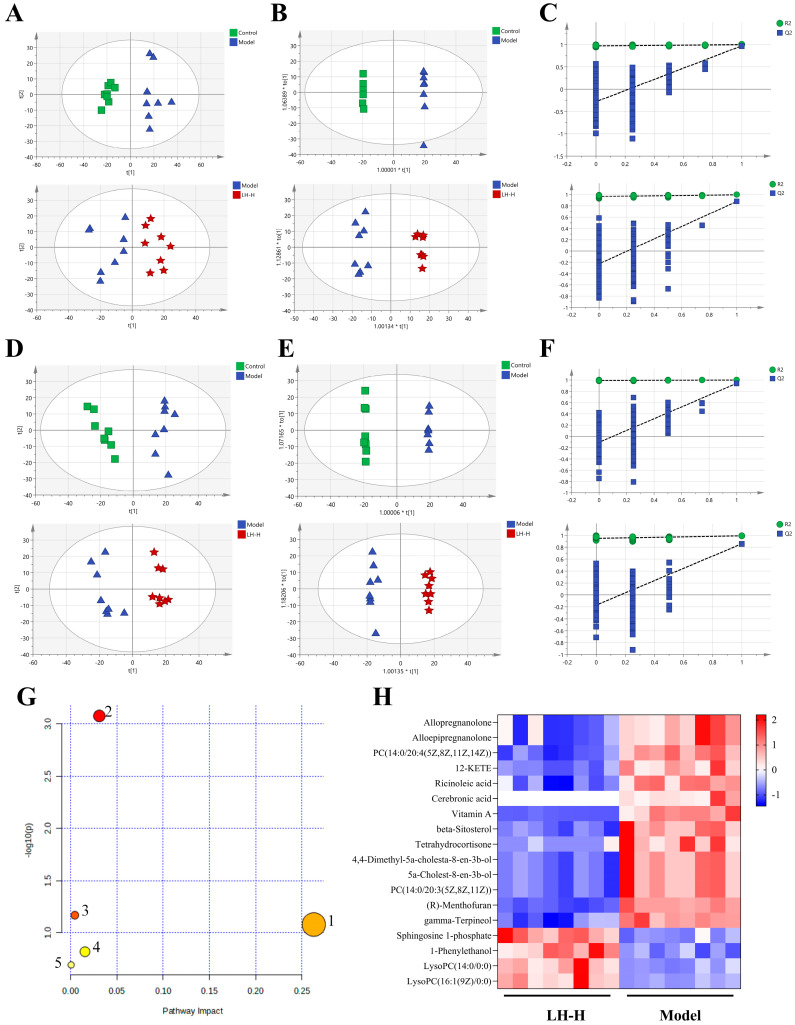
Effects of LH on liver metabolism in T2DM rats. (**A**) Negative ion mode multivariate analyses (PCA). (**B**) Negative ion mode multivariate analyses (OPLS-DA). (**C**) Permutation test of the OPLS-DA model (negative ion mode). (**D**) Positive ion mode multivariate analyses (PCA). (**E**) Positive ion mode multivariate analyses (OPLS-DA). (**F**) Permutation test of the OPLS-DA model (positive ion mode). (**G**) Pathway impact analysis of LH-H treatment: 1, retinol; 2, steroid biosynthesis; 3, steroid hormone biosynthesis; 4, sphingolipid; 5, arachidonic acid metabolism. (**H**) Hepatic metabolite heatmap following LH-H intervention.

PCA and OPLS-DA analyses revealed distinct metabolic profiles among the control, model, and LH-H groups. The score plots demonstrated clear separation between these groups in both positive (Figure 4D,E) and negative modes (Figure 4A,B), indicating that LH-H treatment modulated dysregulated hepatic metabolites. The OPLS-DA model parameters demonstrated excellent fit and predictive ability (Table S3), with model validity confirmed through 200 permutation tests in both ionization modes (Figure 4F, positive mode; Figure 4C, negative mode).

Potential biomarkers were identified, with eighteen metabolites satisfying the screening criteria (Figure 4H and Table 2). Pathway analysis using MetaboAnalyst (version 6.0, https://www.metaboanalyst.ca/, accessed on 19 August 2025) revealed five significantly altered metabolic pathways: retinol metabolism, steroid biosynthesis, steroid hormone biosynthesis, sphingolipid metabolism, and arachidonic acid metabolism. Notably, S1P was identified as a key metabolite in the sphingolipid metabolism pathway.

### 3.6. Effects of LH on the S1P and PI3K/AKT Signaling Pathway in T2DM Rats

**Figure 5:**
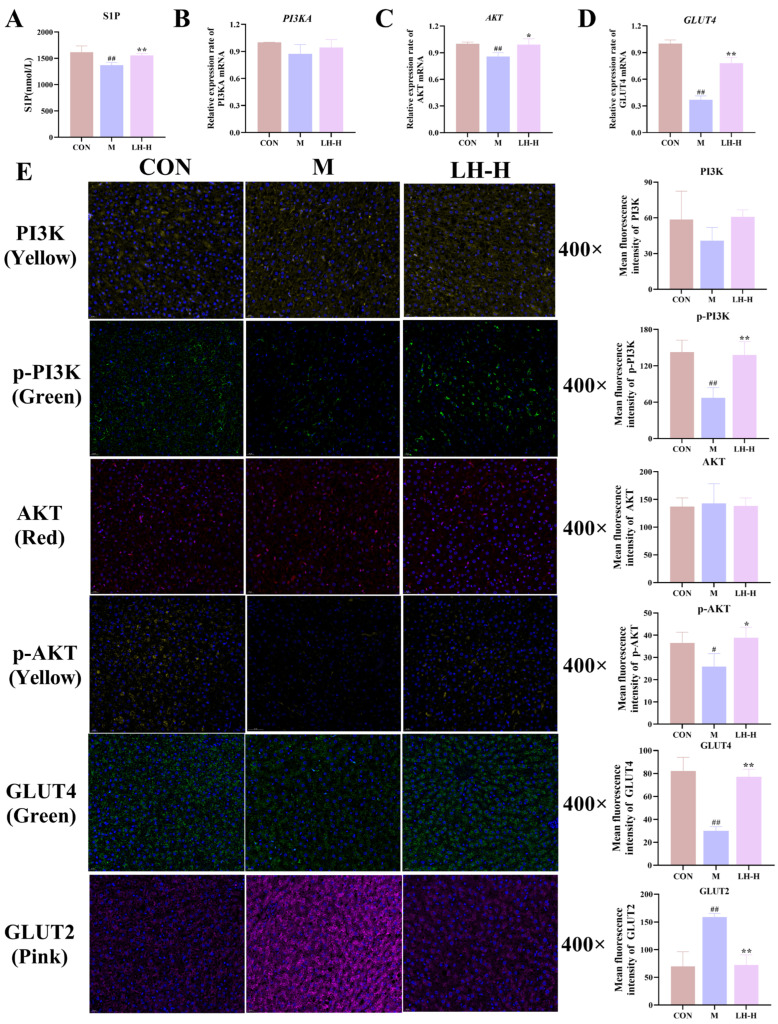
Effects of LH on the S1P and PI3K/AKT signaling pathway in T2DM rats. (**A**) Levels of S1P in T2DM rats’ liver (n = 6). (**B**–**D**) Gene expression levels of PI3K, AKT, and GLUT4 in liver tissues (n = 3). (**E**) Protein expression levels of PI3K, p-PI3K, AKT, p-AKT, GLUT4, and GLUT2 in liver tissue (n = 3). Scale bar = 20 µm. ^#^ *p* < 0.0 ^##^ *p* < 0.01 vs. CON group; * *p* < 0.05, ** *p* < 0.01 vs. M group.

As illustrated in Figure 5, S1P levels were significantly increased in the liver following LH treatment. LH treatment markedly upregulated the expression of phosphorylated PI3K, AKT, and GLUT4 in diabetic rats’ livers, while downregulating the expression of GLUT2. It indicated that LH may improve glucose metabolism in T2DM through the S1P and PI3K/AKT signaling pathways.

### 3.7. Spearman Correlation Matrix of Hepatic Metabolites and Biochemical Parameters

**Figure 6:**
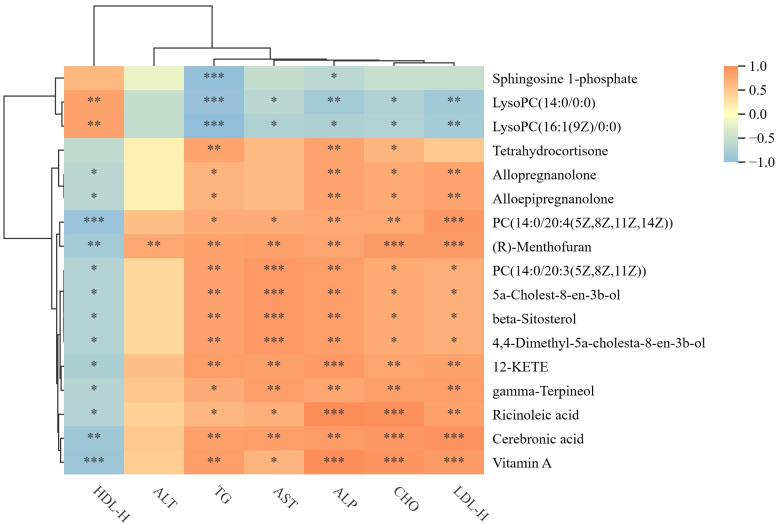
Spearman correlation matrix of hepatic metabolites and biochemical parameters. Biochemical measures (X-axis) and differential liver metabolites (Y-axis) display positive (orange) and negative (blue) associations. The darker the color, the stronger the correlation. * *p* < 0.05, ** *p* < 0.01, *** *p* < 0.001.

The analysis revealed that ALT, AST, ALP, TG, CHO, and LDL-H levels were positively correlated with several metabolites, including tetrahydrocortisone, allopregnanolone, alloepipregnanolone, PC (14:0/20:4 (5Z,8Z,11Z,14Z)), (R)-Menthofuran, PC (14:0/20:3 (5Z,8Z,11Z)), 5a-Cholest-8-en-3b-ol, beta-Sitosterol, 4,4-Dimethyl-5a-cholesta-8-en-3b-ol, 12-KETE, gamma-Terpineol, Ricinoleic acid, Cerebronic acid, and Vitamin A. Conversely, these biochemical markers were negatively correlated with Sphingosine 1-phosphate, LysoPC (14:0/0:0), and LysoPC (16:1(9Z)/0:0). In contrast, HDL-C correlated with individual differential metabolites in contrast to other biochemical indicators. These were shown in Figure 6.

### 3.8. Molecular Modeling and Docking of Active Components of LH with PI3Ka

**Figure 7:**
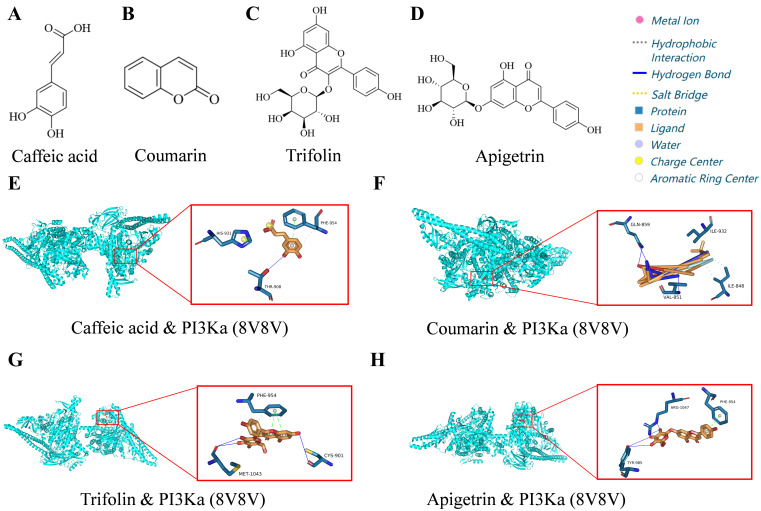
Molecular docking of active components of LH with PI3Ka. (**A**–**D**) Chemical structures of caffeic acid, coumarin, trifolin, and apigetrin. (**E**–**H**) Molecular docking models showing the binding site of these compounds with PI3Ka. This section may be divided by subheadings. It should provide a concise and precise description of the experimental results and their interpretation, as well as the experimental conclusions that can be drawn.

To identify potential bioactive components within LH that improve T2DM, molecular docking analysis was conducted on four selected compounds—caffeic acid, coumarin, trifolin, and apigetrin—with PI3Ka. The results demonstrated that these compounds formed stable interactions with PI3Ka (Table S4 and Figure 7), with binding energies of −6.7 kcal/mol (caffeic acid), −7.3 kcal/mol (coumarin), −11.8 kcal/mol (trifolin), and −11.1 kcal/mol (apigetrin), respectively. These interactions involved metal ion coordination, hydrophobic interactions, and hydrogen bonds. The docking results suggested that these four components may effectively bind to PI3Ka, indicating their potential as active components of LH in modulating PI3K/AKT signaling.

## 4. Discussion

T2DM is characterized by chronic hyperglycemia resulting from insulin resistance and disrupted glycolipid metabolism and is frequently associated with multisystem complications [11]. *Artemisia integrifolia* Linn., known as a green vegetable and functional food, has been reported to possess hypoglycemic and hypolipidemic properties [19], yet its anti-diabetic potential mechanisms remain underexplored. Metformin, a first-line anti-diabetic agent known to enhance insulin sensitivity [20], served as a positive control. LH extract was then evaluated for its therapeutic efficacy in comparison to metformin. Our phytochemical characterization revealed LH extract’s diverse composition, with UPLC-Q-TOF-MS/MS identifying 66 constituents dominated by flavonoids, acids, and coumarins—phytochemical classes widely recognized for metabolic modulation.

Persistent hyperglycemia, body weight loss, and insulin resistance are common manifestations of T2DM, with body weight resulting from increased lipolysis and muscle atrophy [21]. Notably, the observed preservation of body weight and reduction in insulin levels in LH-treated groups points to potential effects on energy partitioning, contrasting the characteristic catabolic state of diabetes. Moreover, liver function markers, including ALT, AST, and ALP, are elevated in diabetes due to hepatocellular injury and increased membrane permeability [22]. LH treatment significantly lowered these enzymes, suggesting its hepatoprotective potential. Histological analysis further confirmed the improvements in hepatic architecture and lipid accumulation in LH-treated rats, indicating its role in reversing T2DM-induced liver damage. The phenomenon proved that LH might have hypoglycemic and anti-insulin resistance effects in T2DM.

In addition to glucose regulation, dyslipidemia is a hallmark of T2DM, often exacerbating hepatic metabolic stress. Diabetic dyslipidemia typically presents with increased levels of TG, CHO, and LDL-C, along with decreased HDL-C level [23]. Correspondingly, the results showed that LH improved serum lipid profiles, reduced TG, CHO, and LDL-C, and increased HDL-C levels. Additionally, hepatic lipid accumulation was markedly reduced, suggesting that LH may enhance hepatic lipid export or processing pathways. Studies have confirmed that LH has a good hypolipidemic effect [14], and these results further verified this conclusion.

To investigate the mechanistic basis of LH’s anti-diabetic effects, we performed hepatic metabolomic profiling to identify critical metabolic nodes influenced by T2DM pathology and LH intervention. 18 potential biomarkers were identified in the liver, categorized into glucose-related compounds (tetrahydrocortisone), lipid compounds (S1P, vitamin A, β-sitosterol, and so on), and glycolipid compounds (cerebronic acid). Following high-dose LH treatment, significant changes in metabolite levels were observed. Among them, S1P, LysoPC (14:0/0:0), and LysoPC (16:1(9Z)/0:0) were upregulated, while tetrahydrocortisone, vitamin A, β-sitosterol, and others were downregulated. Notably, the observed metabolic shifts suggested LH may exert therapeutic effects through multiple coordinated mechanisms. The affected metabolic pathways primarily included retinol metabolism, steroid biosynthesis, steroid hormone biosynthesis, sphingolipid metabolism, and arachidonic acid metabolism, which are all closely linked to glycolipid metabolism regulation. Retinol, obtained from dietary sources such as retinyl esters or provitamin Aβ-carotene, plays a crucial role in maintaining liver function. Disruptions in its metabolism have been associated with liver dysfunction [24]. Similarly, steroid hormones, including glucocorticoids, are key regulators of metabolic homeostasis [25]. Dysregulated glucocorticoid signaling promotes hyperglycemia, dyslipidemia, insulin resistance, and hepatic steatosis—factors that contribute to metabolic syndrome and T2DM pathogenesis [26]. Our findings demonstrated that LH treatment resulted in the upregulation of tetrahydrocortisone levels, indicating that LH may modulate steroid biosynthesis pathways to mitigate T2DM progression. Moreover, S1P, a bioactive lipid, has been recognized for its protective effects against T2DM, including its roles in β-cell proliferation, inhibition of apoptosis, and regulation of insulin sensitivity via adiponectin [27]. In this study, we observed that S1P levels were significantly reduced in T2DM rat livers but were restored after LH treatment. Due to the close relationship between S1P and T2DM, we further investigated the potential pathways associated with S1P in the treatment of T2DM.

Studies have confirmed that S1P activates the PI3K/AKT pathway, promoting cellular growth and inhibiting apoptosis [28]. The PI3K/AKT pathway is a critical regulator of glycolipid metabolism [29], and its dysregulation is a key contributor to the metabolic abnormalities seen in T2DM [30]. Significantly, insulin enhances cellular glucose absorption through PI3K/AKT-mediated translocation of GLUT4 to the plasma membrane [31]. GLUT2 is predominantly localized on hepatocyte plasma membranes and enhances intracellular glucose production during fasting, thereby facilitating glucose efflux [32]. Moreover, GLUT2 primarily facilitates hepatic glucose clearance, whereas GLUT4 exhibits functionally antagonistic effects in glucose homeostasis regulation [11]. It showed that LH not only alleviated the PI3K/AKT signaling pathway but also enhanced GLUT4 translocation and downregulated GLUT2 levels in the liver. It indicated that LH may ameliorate glycolipid metabolic dysregulation in T2DM rats through the S1P and PI3K/AKT signaling axis. LH may exert its hypoglycemic effects through this potential regulatory mechanism. Nevertheless, the potential binding interactions of LH with either S1P or PI3K remain to be experimentally validated through targeted antagonist studies, which constitute an important focus of our future investigations.

Correlation analysis between biochemical indicators and liver metabolites further highlighted the potential of these metabolites as biomarkers for understanding LH’s mechanisms in T2DM treatment. By examining the differential metabolite levels and their association with various biochemical markers, we gained deeper insight into LH’s therapeutic effects.

To explore the material basis for LH’s therapeutic efficacy, we focused on four predominant constituents (caffeic acid, coumarin, trifolin, and apigetrin) identified through chromatographic profiling. Molecular docking analyses demonstrated notable binding interactions with PI3K (binding energies ≤-6 kcal/mol), suggesting they may be the active ingredients responsible for LH’s effects. These findings warrant further investigation into the mechanisms by which these compounds interact with PI3Ka in the context of T2DM treatment. However, it is not known whether these drugs can play a role in the treatment of T2DM, and we will explore their effects and mechanisms in depth in future studies.

## 5. Conclusions

This study provides strong evidence that LH can improve glycolipid metabolism disorders in T2DM by regulating metabolic profiling. We identified 18 potential biomarkers and 5 metabolic pathways that could be involved in the therapeutic action of LH in T2DM. Furthermore, we demonstrated for the first time that LH may alleviate glycolipid metabolic dysregulation in T2DM through the S1P and PI3K/AKT pathways. Additionally, 66 compounds were identified in LH, with four compounds (caffeic acid, coumarin, trifolin, and apigetrin) showing promise as active ingredients that may contribute to the treatment effects. These findings lay a scientific foundation for the use of LH as a novel functional food or dietary supplement for the prevention and treatment of T2DM. Future studies will focus on further investigating the active ingredients in LH and validating its safety and efficacy as a potential food-based treatment for T2DM.

## Figures and Tables

**Table 1:** Primer sequence of genes.

Names	Forward (5′-3′)	Reverse (5′-3′)
*PI3KA*	CTAAGGAGGAGCACTGTCCGTTG	GAGATTCAAAGCCATTTTCCCG
*AKT*	ATCGTGTGGCAAGATGTGTATGAG	GCTGAGTAGGAGAACTGGGGAAA
*GAPDH*	TTCAGCTCTGGGATGACCTT	TGCCACTCAGAAGACTGTGG

Italic font represents each gene name.

**Table 2:** Identified potential biomarkers in the liver regulated by LH-H.

Model	Rt (Time)	Adducts	Measured Mass	Formula Compound	Tentative Identification	HMDB ID	Trend
ESI (+)	13.26	M+H−H_2_O	269.2268	C_20_H_30_O	Vitamin A	HMDB0000305	↓
	13.94	M+H−H_2_O	397.3834	C_29_H_50_O	beta-Sitosterol	HMDB0000852	↓
	5.36	M+H−H_2_O	347.223	C_21_H_32_O_5_	Tetrahydrocortisone	HMDB0000903	↓
	13.94	M+H−H_2_O	397.3834	C_29_H_50_O	4,4-Dimethyl-5a-cholesta-8-en-3b-ol	HMDB0006840	↓
	13.94	M+H−H_2_O	369.3515	C_27_H_46_O	5a-Cholest-8-en-3b-ol	HMDB0006841	↓
	13.99	M+H	756.5545	C_42_H_78_NO_8_P	PC (14:0/20:3 (5Z,8Z,11Z))	HMDB0007881	↓
	6.52	M+H	468.3087	C_22_H_46_NO_7_P	LysoPC (14:0/0:0)	HMDB0010379	↑
	6.84	M+H, M+Na, M+H−H_2_O	494.3237	C_24_H_48_NO_7_P	LysoPC (16:1(9Z)/0:0)	HMDB0010383	↑
	13.33	M+H−H_2_O	133.1013	C_10_H_14_O	(R)-Menthofuran	HMDB0036089	↓
	10.33	2M+H	309.2803	C_10_H_18_O	gamma-Terpineol	HMDB0036993	↓
ESI (−)	6.48	M+FA−H	424.2466	C_18_H_38_NO_5_P	Sphingosine 1-phosphate	HMDB0000277	↑
	11.16	M−H	317.2483	C_21_H_34_O_2_	Allopregnanolone	HMDB0001449	↓
	11.16	M−H	317.2483	C_21_H_34_O_2_	Alloepipregnanolone	HMDB0001455	↓
	10.64	M−H	752.523	C_42_H_76_NO_8_P	PC (14:0/20:4 (5Z,8Z,11Z,14Z))	HMDB0007883	↓
	10.73	M−H	317.213	C_20_H_30_O_3_	12-KETE	HMDB0013633	↓
	12.36	M−H	121.0655	C_8_H_10_O	1-Phenylethanol	HMDB0032619	↑
	11.71	M−H	297.2431	C_18_H_34_O_3_	Ricinoleic acid	HMDB0034297	↓
	10.95	M−H_2_O−H	365.3423	C_24_H_48_O_3_	Cerebronic acid	HMDB0039540	↓

Arrows in trend indicated the changing information of differential metabolites in LH-H groups compared with model group.

## Data Availability

The original contributions presented in the study are included in the article/Supplementary Materials, further inquiries can be directed to the corresponding authors.

## Supplementary Materials

The following supporting information can be downloaded at: https://www.mdpi.com/article/10.3390/foods14172945/s1, Figure S1: total ion current chromatograms of LH in the positive (POS) and negative (NEG) ionization modes; Table S1: characterization of the chemical composition of LH extraction components in positive ion mode; Table S2: characterization of the chemical composition of LH extraction components in negative ion mode; Table S3: OPLS-DA model parameters; Table S4: molecular docking score.

## Abbreviations

AA	arachidonic acid
AKT	protein kinase B
ALP	alkaline phosphatase
ALT	alanine aminotransferase
AST	aspartate aminotransferase
CHO	cholesterol
ESI	electrospray ionization source
FBG	fasting blood glucose
GAPDH	glyceraldehyde-3-phosphate dehydrogenase
GLU	glucose
GLUT4	glucose transporter protein 4
HDL-C	high density lipoprotein cholesterol
H&E	haematoxylin and eosin
LDL-C	low density lipoprotein cholesterol
LH	*Artemisia integrifolia* Linn.
Met	metformin
OPLS-DA	orthogonal partial least-squares-discriminant analysis
ORO	Oil red O
PCA	principal component analysis
PI3K	phosphatidylinositol 3-kinase
QC	quality control
RT-qPCR	quantitative real-time PCR
S1P	sphingosine 1-phosphate
SOD	superoxide dismutase
SPF	specific pathogen-free
STZ	streptozotocin
T2DM	Type II diabetes mellitus
TCM	Traditional Chinese Medicine
TG	triglycerides
VIP	variable importance in projection
