# Supplementary material for: An Extensive Analysis of Artemisia integrifolia Linn. on T2DM: Investigating Glycolipid Metabolism, Metabolic Profiling, and Molecular Docking for Potential Functional Food Applications
Source: Foods. 2025 Aug 24;14(17):2945. doi: 10.3390/foods14172945 (PMC12428366; doi:10.3390/foods14172945)
Supplement: Supplementary file 1 [file foods-14-02945-s001.zip › foods-3793581-supplementary.pdf]

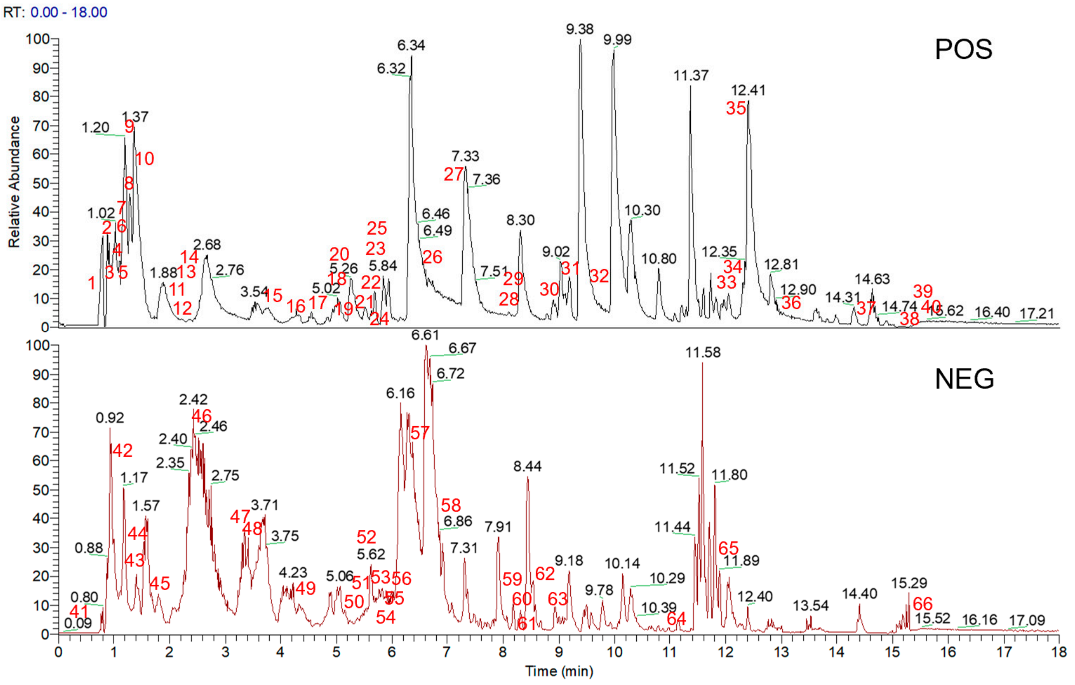

Figure S1. Total ion current chromatograms of LH in the Positive (POS) and negative (NEG) ionization modes.

Table S1 Characterization of the chemical composition of LH extraction components in positive ion mode.

NO	t_R (min)	Formula	Name	Ion condition	Neuctral Mass (Da)	Observed (m/z)	MS/MS fragment	Type
1	0.81	$C_6H_{14}N_4O_2$	DL-Arginine	M+H	174.11	175.1175	70, 60, 175, 116	Carboxylic acids
2	0.885	$C_5H_{13}NO$	Choline	M+H	103.1	104.1063	104, 60	Organonitrogen
3	0.913	$C_5H_9NO_2$	L-Proline	M+H	115.06	116.0697	70, 116, 71	Carboxylic acids
4	1.034	$C_5H_5N_5$	Adenine	M+H	135.05	136.0606	119	Imidazopyrimidines

5	1.113	$C_6H_5NO_2$	Nicotinic acid	M+H	123.03	124.0383	80, 106, 78	Pyridines
6	1.13	$C_9H_{11}NO_3$	L-Tyrosine	M+H	181.07	182.0797	136, 123, 119, 165, 91, 95	Carboxylic acids
7	1.133	$C_{10}H_{13}N_5O_4$	Adenosine	M+H	267.09	268.1012	136, 268	Purine nucleosides
8	1.322	$C_5H_7NO_3$	L-Pyroglutamic acid	M+H	129.04	130.0489	84, 70, 130, 56	Carboxylic acids
9	1.368	$C_9H_{11}NO_2$	L-Phenylalanine	M+H	165.08	166.0847	120, 103, 91	Carboxylic acids
10	1.497	C_5H_9NO	N-Methyl-2-pyrrolidone	M+H	99.07	100.0751	100, 82	Pyrrolidines
11	2.214	$C_6H_6O_3$	Maltol	M+H	126.03	127.038	127, 109, 53, 81, 55	Pyrans
12	2.231	$C_6H_6O_4$	Kojic acid	M+H	142.03	143.0327	143, 69, 125	Pyrans
13	2.261	$C_{11}H_{12}N_2O_2$	DL-Tryptophan	M+H	204.09	205.0953	118, 144, 132, 159	Indoles

14	2.652	$C_6H_{11}NO$	Caprolactam	M+H	113.08	114.0905	114, 96, 55, 72	Lactams
15	3.576	$C_9H_6O_4$	Esculetin	M+H	178.03	179.0324	179, 123, 151, 133	Coumarins
16	4.024	$C_{10}H_9N$	6-Methylquinoline	M+H	143.07	144.0795	144, 70, 98, 117, 84	Quinolines
17	4.691	$C_{12}H_{18}O_3$	Jasmonic acid	M+H	210.12	211.1309	105, 147	Fatty Acyls
18	5.008	$C_{11}H_8N_2$	Norharman	M+H	168.07	169.0746	169	Indoles
19	5.18	$C_8H_8O_3$	Vanillin	M+H	152.05	153.0533	93, 125, 153	Phenols
20	5.182	$C_{10}H_{10}O_2$	Methyl cinnamate	M+H	162.07	163.074	113, 103,163,91	Cinnamic acids
21	5.447	$C_{13}H_{13}N_3$	N, N'-Diphenylguanidine	M+H	211.11	212.1163	94	Benzene
22	5.6	$C_{10}H_{16}O$	D- (+)-Camphor	M+H	152.12	153.1261	153, 109	Prenol lipids
23	5.686	$C_9H_{10}O_2$	4'-Methoxyacetophenone	M+H	150.07	151.074	151	Organooxygen

24	5.728	$C_{21}H_{20}O_{10}$	Vitexin	M+H	432.1	433.1089	283, 313, 397, 415	Flavonoids
25	5.73	$C_{10}H_8O_4$	7-hydroxy-6-methoxy-2H-chromen-2-one	M+H	192.04	193.0481	193, 133, 137	Coumarins
26	6.577	$C_{21}H_{20}O_{10}$	Apigetrin	M+H	432.1	433.1089	119	Flavonoids
27	7.324	$C_9H_6O_2$	Coumarin	M+H	146.04	147.0427	147, 91, 65, 119	Coumarins
28	8.019	$C_{16}H_{12}O_7$	Isorhamnetin	M+H	316.06	317.0623	302, 274, 153	Flavonoids
29	8.808	$C_9H_{14}O$	Isophorone	M+H	138.1	139.1105	139, 93, 121	Organooxygen
30	8.835	$C_{15}H_{10}O_5$	Genistein	M+H	270.05	271.0575	153, 243	Isoflavonoids
31	9.188	$C_{15}H_{24}O$	(-)-Caryophyllene oxide	M+H	220.18	221.188	147, 95, 109, 81, 161	Prenol lipids
32	9.674	$C_{15}H_{12}O_4$	Isoliquiritigenin	M+H	256.07	257.0784	137, 239	Linear 1,3-diarylpropanoids
33	12.232	$C_{18}H_{30}O_3$	9-Oxo-10(E),12(E)-	M+H	294.22	295.2232	277, 81, 151, 69, 95	Fatty Acyls

			octadecadienoic acid					
34	12.284	$C_{18}H_{37}NO$	Stearamide	M+H	283.29	284.2924	284, 88, 102, 116	Carboximidic acids
35	12.405	$C_{16}H_{22}O_4$	Dibutyl phthalate	M+H	278.15	279.1557	93	Benzene
36	12.907	$C_{20}H_{37}NO_2$	Linoleoyl ethanolamide	M+H	323.28	324.287	324, 109	Organonitrogen
37	14.503	$C_{18}H_{35}NO$	Oleamide	M+H	281.27	282.2766	282, 83, 97, 247	Fatty Acyls
38	15.237	$C_9H_{10}N_2$	5,6-Dimethylbenzimidazole	M+H	146.08	147.0905	146	Benzimidazoles
39	15.406	$C_8H_{11}N$	2,6-Xylidine	M+H	121.09	122.0956	122, 72, 107	Benzene
40	15.409	$C_7H_{19}N_3$	Spermidine	M+H	145.16	146.164	72, 112, 558, 146	Organonitrogen

Table S2 Characterization of the chemical composition of LH extraction components in negative ion mode.

NO	t_R (min)	Formula	Name	Ion condition	Neuctral Mass (Da)	Observed (m/z)	MS/MS fragment	Type
41	0.025	$C_7H_6O_3$	Salicylic acid	M-H	138.03	137.022	137, 93, 92, 108	Benzene
42	0.927	$C_7H_{12}O_6$	D- (-)-Quinic acid	M-H	192.06	191.0536	191, 85, 127, 111	Organooxygen
43	1.485	$C_8H_8O_4$	Homogentisic acid	M-H	168.04	167.0325	123, 167, 139	Benzene
44	1.514	$C_7H_6O_4$	2,3-Dihydroxybenzoic acid	M-H	154.02	153.0169	153, 109	Benzene
45	1.772	$C_7H_{12}O_4$	Pimelic acid	M-H	160.07	159.0639	159, 57, 97, 115	Fatty Acyls
46	2.436	$C_{16}H_{18}O_9$	Chlorogenic acid	M-H	354.09	353.083	191,135,161	Organooxygen
47	3.493	$C_8H_8O_3$	4-Anisic acid	M-H	152.04	151.0376	151	Benzene
48	3.687	$C_9H_8O_4$	Caffeic acid	M-H	180.04	179.0325	135, 179,134	Cinnamic acids

49	4.389	$C_7H_6O_2$	Benzoic acid	M-H	122.03	121.0271	93	Benzene
50	5.532	$C_9H_{10}O_3$	3-Phenyllactic acid	M-H	166.06	165.0533	147, 119, 165, 72	Phenylpropanoic acids
51	5.589	$C_9H_6O_3$	4-Hydroxycoumarin	M-H	162.03	161.0219	161,133	Coumarins
52	5.617	$C_{27}H_{30}O_{16}$	Rutin	M-H	610.15	609.1403	300, 271, 301	Flavonoids
53	5.668	$C_{10}H_{10}O_4$	trans-Ferulic acid	M-H	224.07	193.0481	134, 149,193,135,	Cinnamic acids
54	5.803	$C_{21}H_{18}O_{13}$	Miquelianin	M-H	478.07	477.0634	301, 477	Flavonoids
55	5.934	$C_{15}H_{10}O_6$	Kaempferol	M-H	286.05	285.0382	285, 151	Flavonoids
56	5.942	$C_{21}H_{20}O_{11}$	Cynaroside	M-H	448.1	447.0894	285	Flavonoids
57	6.426	$C_{21}H_{20}O_{11}$	Trifolin	M-H	448.1	447.0892	285	Flavonoids
58	6.854	$C_9H_{16}O_4$	Azelaic acid	M-H	188.1	187.0951	125, 187, 97, 123, 57, 169	Fatty Acyls

59	7.963	$C_{15}H_{10}O_6$	Luteolin	M-H	286.05	285.0385	285,133,151,175,199	Flavonoids
60	7.977	$C_{15}H_{10}O_7$	Quercetin	M-H	302.04	301.0334	151, 178	Flavonoids
61	8.025	$C_{16}H_{12}O_7$	Isorhamnetin	M-H	316.06	315.0492	300	Flavonoids
62	8.657	$C_{10}H_{10}O_4$	trans-Ferulic acid	M-H	194.06	193.0482	149,134	Cinnamic acids
63	8.838	$C_{15}H_{10}O_5$	Apigenin	M-H	270.05	269.0439	151, 225	Flavonoids
64	10.951	$C_{15}H_{10}O_4$	Chrysin	M-H	254.06	253.0487	209	Flavonoids
65	11.869	$C_{18}H_{28}O_3$	12-oxo Phytodienoic Acid	M-H	292.2	291.1946	291, 247, 273	Fatty Acyls
66	15.408	$C_{18}H_{32}O_2$	Linoleic Acid	M-H	280.24	279.2308	235	Fatty Acyls

Table S3 OPLS-DA model parameters.

	CON VS M (ESI-)	CON VS M (ESI+)	M VS LH-H (ESI-)	M VS LH-H (ESI+)
R^2X	0.468	0.41	0.397	0.294
R^2Y	0.999	0.999	0.998	0.992
Q^2	0.973	0.942	0.877	0.858

Table S4 Molecular docking score.

	Trifolin	Apigetrin	Coumarin	Caffeic acid

PI3Ka (8V8V)	-11.8	-11.1	-7.3	-6.7
